# *MYH6* Variants Are Associated with Atrial Dysfunction in Neonates with Hypoplastic Left Heart Syndrome

**DOI:** 10.3390/genes15111449

**Published:** 2024-11-10

**Authors:** Melissa Quintanilla Anfinson, Sara Creighton, Pippa M. Simpson, Jeanne M. James, Phoebe Lim, Peter C. Frommelt, Aoy Tomita-Mitchell, Michael E. Mitchell

**Affiliations:** 1 Department of Surgery, Medical College of Wisconsin, Milwaukee, WI 53226, USApjlim@mcw.edu (P.L.); amitchell@mcw.edu (A.T.-M.); 2 Division of Pediatric Cardiology, Department of Pediatrics, Children’s Wisconsin, Milwaukee, WI 53226, USA; screighton@childrenswi.org (S.C.); pfrommelt@childrenswi.org (P.C.F.); 3 Division of Quantitative Health Sciences, Department of Biostatistics, Medical College of Wisconsin, Milwaukee, WI 53226, USA; psimpson@mcw.edu; 4 Department of Pediatrics, Children’s Mercy Kansas City, Kansas City, MO 64108, USA; jmjames2@cmh.edu; 5 Division of Congenital Cardiac Surgery, Divisions of Thoracic and Cardiac Surgery, Department of Surgery, Children’s Wisconsin, Milwaukee, WI 53226, USA

**Keywords:** congenital heart disease, hypoplastic left heart syndrome, *MYH6*, atrial strain, speckle-tracking echocardiography

## Abstract

**Background**: *MYH6* variants are the most well-known genetic risk factor (10%) for hypoplastic left heart syndrome (HLHS) and are associated with decreased cardiac transplant-free survival. *MYH6* encodes for α-myosin heavy chain (α-MHC), a contractile protein expressed in the neonatal atria. We therefore assessed atrial function in HLHS patients with *MYH6* variants. **Methods**: We performed a retrospective, blinded assessment of pre-stage I atrial function using 2D speckle-tracking echocardiography (2D-STE). Variant carriers were control-matched based on AV valve anatomy, sex, and birth year. Studies were obtained postnatally from awake patients prior to surgical intervention. Right atrial (RA) and right ventricular (RV) strain and strain rate (SR) were measured from the apical four-chamber view. **Results**: A total of 19 HLHS patients with *MYH6* variants had echocardiograms available; 18 were matched to two controls each, and one had a single control. RA active strain (ASct) was decreased in variant carriers (−1.41%, IQR −2.13, −0.25) vs. controls (−3.53%, IQR −5.53, −1.28; *p* = 0.008). No significant differences were identified in RV strain between the groups. RA reservoir strain (ASr) and conduit strain (AScd) positively correlated with heart rate (HR) in *MYH6* variant carriers only (ASr R = 0.499, *p* = 0.029; AScd R = 0.469, *p* = 0.043). RV global longitudinal strain (GLS) as well as RV systolic strain (VSs) and strain rate (VSRs) correlated with HR in controls only (GLS R = 0.325, *p* = 0.050; VSs R = 0.419, *p* = 0.010; VSRs R = 0.410, *p* = 0.012). **Conclusions**: We identified functional consequences associated with *MYH6* variants, a known risk factor for poor outcomes in HLHS. *MYH6* variant carriers exhibit impaired RA contractility despite there being no differences in RV function between variant carriers and controls. *MYH6* variants are also associated with an ineffective RA reservoir and conduit function at high heart rates, despite preserved RV diastolic function. RA dysfunction and reduced atrial “kick” may therefore be a significant contributor to RV failure and worse clinical outcomes in HLHS patients with *MYH6* variants.

## 1. Introduction

Hypoplastic left heart syndrome (HLHS) is a severe and complex congenital heart disease which is uniformly fatal early in life without surgical intervention. A three-stage surgical palliation, which is now the standard of care, allows many patients to live into adulthood. Despite these advances, many risk factors have been identified that affect both short- and long-term survival, including obstructive pulmonary venous return, restrictive or intact atrial septum, coronary artery abnormalities, tricuspid regurgitation, smaller ascending aorta size, premature birth, and HLHS associated with a genetic syndrome [1,2]. Surgical intervention for HLHS is palliative in nature, with many patients ultimately requiring heart transplantation. Although the cumulative risk of transplantation becomes higher as time goes on, transplant need can arise at any time following birth and at any stage of surgical reconstruction [3,4,5]. The identification of additional predictors that improve risk stratification would help inform clinical decisions regarding if, and when, to list a patient for cardiac transplant.

We previously reported that rare, predicted damaging variants in the *MYH6* gene were enriched in HLHS and associated with reduced cardiac transplant-free survival [6]. These findings were supported by our analysis of a control-matched subset [7]. We therefore sought to identify measurable functional consequences of *MYH6* variants that could aid in improved transplant risk stratification of these patients, using more recent survival and imaging data from previously published cohorts [6,7] along with additional patients that have presented to our institution over the last five years.

*MYH6* encodes for α-myosin heavy chain (α-MHC), a key force-generating protein in cardiac muscle. *MYH6* is expressed in the early developing heart and localizes predominantly to the atria during the first trimester of gestation [8]. Postnatal ventricles express primarily *MYH7*, which encodes for the β isoform of myosin heavy chain (β-MHC). Although HLHS is characterized by diminished ventricular size, the formation of the heart chambers is flow-mediated [9,10], and atrial contraction is thus essential for proper ventricular development. 

*MYH6* continues to be the predominant isoform expressed in the atria postnatally, suggesting that *MYH6* variants would continue to impact atrial function after birth. The need for transplant is primarily based on the right ventricular (RV) and tricuspid valve function, with consideration given to signs of end-organ damage, including renal disease, protein-losing enteropathy, and plastic bronchitis [3,4,11]. The functional status of the right atrium (RA) is not typically considered, even though the atria play multiple roles in cardiac output. During ventricular systole, the atria collect venous blood flow (‘reservoir’ function), then during early diastole they act as conduits for venous blood in transit to the ventricles (‘conduit’ function). In late ventricular diastole, the atria actively contract (atrial “kick”) to maximize the ventricular end diastolic volume, contributing up to 20–30% of cardiac output [12]. In patients with impaired ventricular function, including single-ventricle patients, the diastolic ventricular filling becomes more reliant on atrial kick. 

We hypothesized that *MYH6* impairs RA contractility, ultimately resulting in decreased RV output and accelerating the failure of surgically-reconstructed HLHS hearts. We further hypothesized that RA dysfunction would present early, prior to detectable RV failure. To test this, we performed a retrospective echocardiogram analysis of pre-surgical RA function in HLHS patients with and without *MYH6* variants. Atrial function was assessed via myocardial strain using semi-automated two-dimensional speckle-tracking echocardiography (2D-STE). Myocardial strain is a measure of tissue deformation and is defined as a change in myocardial fiber length relative to the baseline. 2D-STE calculates myocardial movement via ‘speckles’, naturally occurring acoustical markers in ultrasound. The movement of speckles within a tissue from one echo frame to the next allows them to be translated into myocardial velocity vectors [13].

**Table 1:** Characteristics of *MYH6* variants included in the study. Allele frequencies were obtained from the Genome Aggregation Database Genomes dataset (gnomAD) v4.1.0. Combined annotation-dependent depletion (CADD) scores are based on genome build GRCh38 (CADD v1.7).

Study ID	*MYH6* Variant	dbSNP ID	Allele Frequency	CADD	Previously Reported
R0735	Q277H	rs140660481	2.6 × 10^−4^	25.0	Y [6,14,15]
21_124	A336G	rs138572790	6.8 × 10^−5^	23.7	N
07_155	D383N	N/A	Not reported	25.7	Y [6]
10_121	S385L	rs778319108	5.9 × 10^−5^	24.1	Y [6]
10_121	M436V	N/A	6.2 × 10^−7^	24.9	Y [6]
07_067	R443P	N/A	Not reported	28.7	Y [6,16]
R_0622	F469V	N/A	Not reported	26.0	N
18_001	K867R	rs143284278	8.7 × 10^−6^	24.5	N
07_074	K849-	N/A	N/A	N/A	Y [6]
R0121	A936V	rs199838024	2.7 × 10^−4^	24.6	Y [6,17]
10_249, 09_299	A964S	rs144907522	2.2 × 10^−4^	24.7	Y [6]
07_082	R1151Q	rs745406670	2.7 × 10^−5^	28.0	Y [6]
09_103	A1298V	rs368588052	1.4 × 10^−4^	25.9	Y [6]
09_152, 12_093	T1379M	rs145611185	8.6 × 10^−4^	31	Y [6,18,19,20]
11_003	A1443D	rs727503234	2.0 × 10^−4^	26.2	Y [6,14,19,21]
12_234	E1503V	N/A	Not reported	35.0	Y [6]
09_204	E1584K	rs1280321639	2.5 × 10^−6^	28.7	Y [6]
07_026	E1754X	rs372270600	1.9 × 10^−6^	45.0	Y [6]
R0300	K1840R	rs373629059	1.1 × 10^−4^	28.2	Y [6,22]

## 2. Materials and Methods

### 2.1. Study Population

The cohort was retrospectively selected from all consenting patients with a primary diagnosis of HLHS who were seen at our institution prior to stage I palliation between the years 2000 and 2021. They all had whole-genome or whole-exome sequencing data available. Patients were first identified who carried rare, predicted damaging *MYH6* variants, as defined by minor allele frequency <0.001 in both the Genome Aggregation Database Genomes dataset (gnomAD; v4.1.0) [23] and the Allele Frequency Aggregator dataset (ALFA; v20201027095038), as well as either a scaled combined annotation-dependent depletion (CADD) score >23.0 (GRCh38, v1.7) [24] or a prediction of “damaging” or “probably damaging” by either SIFT [25] or PolyPhen2 [26]. The variant information is summarized in Table 1. All known familial inheritance information concerning these variants has been previously published [6]. Three variants—*MYH6*^R443P^ [6], *MYH6*^E1503V^ [27], and *MYH6*^E1584K^ [28]—segregate with congenital heart disease (CHD), including HLHS, in families. 

*MYH6* variant carriers were matched to two controls based on aortic and mitral valve anatomy, sex when possible, era (i.e., year of birth), and stage I palliation surgical shunt type to allow for future longitudinal comparisons. Controls were selected from all the available HLHS patients with whole-genome or whole-exome sequencing showing the absence of any rare, predicted damaging *MYH6* variant. Patients were excluded who had any chromosomal abnormality or who carried rare, predicted damaging variants in the *MYH7* or *DMD* genes, as well as patients who had received any fetal intervention. All the available data from each patient’s electronic medical record were independently reviewed and verified by two separate individuals. Earlier outcomes for some patients in this cohort have been previously reported [6,7].

### 2.2. Echocardiogram Acquisition

All the echocardiograms were assessed retrospectively. Studies were obtained from awake patients shortly after birth, prior to any surgical intervention. The echocardiogram clip duration and frame rates varied based on age and quality of study.

### 2.3. Myocardial Strain

A 2D-STE analysis was completed on previously existing echocardiograms using TomTec Arena v4.20 (Philips Ultrasound Inc., Reedsville, PA, USA). For multi-beat clips with accompanying electrocardiograms (ECGs), TomTec automatically marked the cardiac cycle, with end-systole positioned at the completion of the T wave and end-diastole at the peak of the R wave. For single-beat clips and those without ECGs of sufficient quality, users manually set end-systole and end-diastole at the opening and closure of the AV valves, respectively. This method is consistent with the recommendations outlined in the most recent European Association of Cardiovascular Imaging (EACVI)/American Society of Echocardiography (ASE)/Industry Task Force to standardize deformation imaging consensus statements [29]. 

All the measurements were obtained from the apical four-chamber view using a semi-automated tracking method. Users first delineated the endocardial border at end-diastole and the TomTec tracking algorithm was allowed to calculate myocardial deformation. The accuracy of the tracking was reviewed by a pediatric cardiology faculty member and if needed, the borders were adjusted manually. Global longitudinal strain (GLS) and time-plots for strain and strain rate (SR) were reported by TomTec.

**Figure 1:**
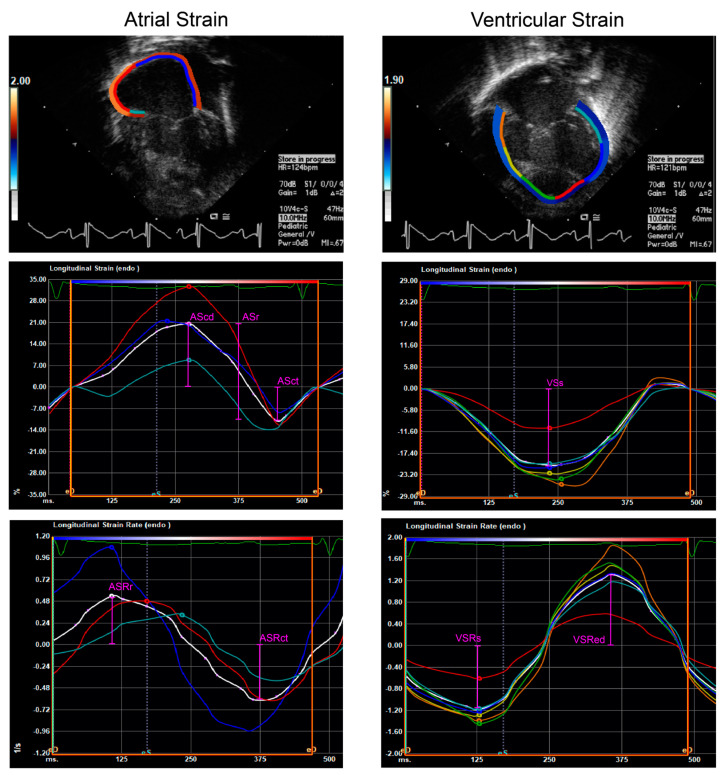
Representative tracings and time-rate plots of atrial and ventricular myocardium in postnatal HLHS. AScd, conduit atrial strain; ASr, reservoir atrial strain; ASct, active/contractile atrial strain; ASRr, reservoir atrial strain rate; ASRct, active/contractile atrial strain rate; VSs, systolic ventricular strain; VSRs, systolic ventricular strain rate; and VSRed, early diastolic ventricular strain rate.

Atrial tracings extended upward from the lateral tricuspid valve annulus, across the roof of the atrium and returning to the septal annulus (Figure 1). Tracings excluded the atrial appendage and, as others have described [30], continued straight across the atrial septal defects present in most HLHS patients. From the atrial strain time-plots, strain during the conduit phase (AScd) was recorded as peak positive strain, strain during the contraction phase (ASct) was recorded as peak negative strain, and strain during the reservoir phase (ASr) was calculated as the difference between the two (AScd–ASct). From the atrial SR time-plots, SR during the contraction phase (ASRct) was recorded as peak negative SR, while SR during the reservoir phase (ASRr) was recorded as peak positive SR. Atrial SR during the conduit phase (ASRcd) was not obtained; as reported by others, two distinct negative peaks are not seen on the SR time-plots in neonates due to high heart rates and limited passive ventricular filling time [31].

Ventricular tracings extended from the lateral tricuspid valve annulus, along the lateral free wall to the RV apex and across the interventricular septum to the septal annulus (Figure 1). Ventricular strain time-plots allowed for the measurement of systolic ventricular strain (VSs), recorded as peak negative strain. Systolic ventricular SR (VSRs), recorded as peak negative SR, and ventricular SR during early diastole (VSRed), recorded as peak positive SR, were obtained from ventricular SR time-plots.

### 2.4. Statistical Analysis

Poor outcomes were defined as the need for mechanical circulatory support, cardiac transplant, or death. Categorical variables are reported as N(%) and groups compared using an exact Fisher test. Continuous variables are reported as median (IQR), unless otherwise indicated, and compared using exact two-tailed nonparametric Mann-Whitney tests. Pearson correlation coefficients were calculated for the linear association of the strain parameters with heart rate, and two-tailed significance was obtained. A general linear model with a normal error link was used to look further at associations between strain and heart rate and the R^2^ was adjusted for these two variables. The 15-year event-free survival between the groups was compared using Kaplan-Meier curves, and the significance was evaluated using a logrank test. For all the analyses, *p* < 0.05 was considered significant but given the small sample size, we considered *p* < 0.1 to be a trend towards significance. No adjustment for multiple comparisons was made when examining our primary outcome of interest (ASct).

## 3. Results

### 3.1. Characteristics of the Study Cohort

A total of 19 HLHS patients carrying *MYH6* variants had echocardiograms available. Some 18 of these patients were matched to two controls each, while one variant carrier had a single control with an echocardiogram available for analysis. Given that the cases and controls were closely matched during the study design, the percentages of each sex, anatomical subtype, and stage I surgical shunt type were similar in each group (Table 2). While we were unable to match directly for the presence of restrictive or intact atrial septum, the overall percentages were similar between the groups (Table 2). 

To the extent possible in a retrospective study, controls were selected that were born in similar eras as variant carriers; the median time between the date of birth of cases and matched controls was 1.58 years (IQR 0.83, 2.42). The median age at the time of the pre-surgical echocardiogram was 0.0 days for both variant carriers (IQR 0.0, 2.0; maximum 7.0) and controls (IQR 0.0, 1.0; maximum 13.0). Most patients were in sinus rhythm at the time of the echocardiograms; a junctional rhythm was suspected in one patient but could not be confirmed. Most had heart rates within expected ranges for the newborn period (120–190 bpm); one was slightly bradycardic (102 bpm).

The majority of the patients in both groups were not mechanically ventilated at the time of the echocardiograms (Table 2). Five (26.3%) *MYH6* variant patients were intubated and mechanically ventilated at the time of imaging. An additional two (10.5%) *MYH6* variant patients required intubation pre-stage I; however, records do not indicate whether this was prior to their echocardiograms. Seven control patients (18.9%) were intubated and mechanically ventilated at the time of imaging, though one of these patients was recorded as being “electively” intubated for transport. One control patient (2.7%) did not have records available stating the mode of breathing.

### 3.2. Event-Free Survival

**Figure 2:**
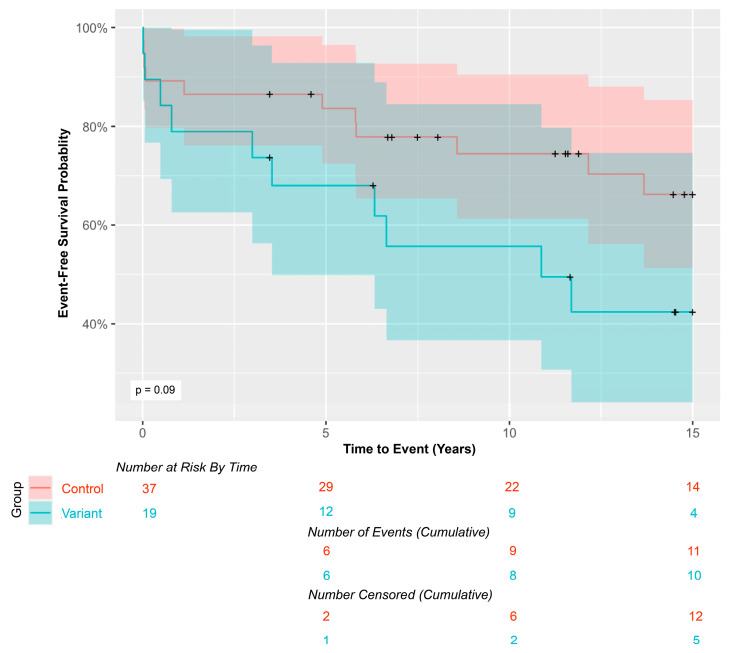
Kaplan-Meier curves comparing 15-year event-free survival in *MYH6* variant carriers vs. controls. Shaded areas represent 95% confidence intervals. ‘+’ signs indicate censored patients.

The 15-year event-free survival analysis of this matched cohort showed a trend of decreased event-free survival in *MYH6* variant carriers (*p* = 0.094, Figure 2). The median follow-up time for patients who did not meet the primary endpoint was 14.5 years (IQR 10.5, 18.5) for the overall cohort, 15.1 years (IQR 10.4, 18.0) for controls, and 14.4 years (IQR 10.8, 18.5) for *MYH6* variant carriers. 

### 3.3. Right Atrial and Ventricular Strain Analyses

The absolute values of RA ASct were significantly decreased in *MYH6* variant carriers (−1.41%, IQR −2.13, −0.25) compared to controls (−3.53%, IQR −5.53, −1.28, *p* = 0.008; Table 3). RA ASRr also trended lower in variant carriers (1.06, (0.78, 1.43)%/s) compared to controls (1.23 (1.05, 1.55)%/s). While this difference did not reach statistical significance (*p* = 0.096), the small sample size suggests further investigation is needed in a larger cohort. No differences were found in RA GLS, AScd, ASr, or ASRct between the groups, and no differences were found in any RV strain indices between the groups. 

We identified no significant relationships between heart rate (HR) and RA GLS, RA ASct, RA ASRr, or RA ASRct in either group (Table 4). Significant positive correlations were found between HR with both RA AScd and RA ASr in *MYH6* variant carriers only. In the general linear model with RA AScd as the outcome, RA AScd was significantly associated with the presence of an *MYH6* variant (*p* = 0.031) and the interaction of a variant differed based on HR (*p* = 0.033) with an adjusted R^2^ = 0.035. This indicates that not only does the presence of an *MYH6* variant have an effect on AScd, but that this effect varies with HR. We similarly assessed the relationship between RV strain indices and HR. Significant positive correlations were found between GLS, VSs, and VSRs with HR in controls; however, we found no significant relationships in *MYH6* variant carriers (Table 4).

## 4. Discussion

### 4.1. Right Atrial Contractile Function

In this study, we identified functional consequences associated with *MYH6* variants, which are a known risk factor for decreased cardiac transplant-free survival in HLHS. Specifically, HLHS patients carrying *MYH6* variants have lower RA ASct vs controls, indicating significantly impaired RA contractility (i.e., decreased atrial “kick”) in the absence of RV dysfunction. This suggests that reduced atrial “kick” may be a significant contributor to RV failure in surgically palliated HLHS patients with *MYH6* variants. These findings are consistent with the predominant atrial localization of α-MHC and its function as a contractile protein, as well as mechanistic studies by our group and others showing impaired cardiomyocyte contractility associated with *MYH6* variants [16,32,33]. 

### 4.2. Right Atrial Conduit and Reservoir Function

Previous characterization of pediatric atrial strain and SR indices report that AScd has an inverse relationship with HR [31]. Conversely, we found that in *MYH6* variant carriers RA conduit and reservoir strain increased as HR increased, indicating RA distension both during atrial filling and during early ventricular filling after the opening of the tricuspid valve. Notably, we did not find statistically significant differences in RA reservoir or conduit strain when heart rate is not taken into account. When considering this in the context of impaired atrial contraction, we hypothesize that RA distension is secondary to the inability of the RA to increase its contractile activity and compensate for decreased passive ventricular filling time as HR increases. Given that single-ventricle patients are more reliant on the atrial contribution to ventricular filling when compared to healthy pediatric patients [30], impaired atrial contraction in *MYH6* variant carriers is likely to be especially detrimental for cardiac function in this population. 

### 4.3. Impact of Right Ventricular Function

It is important to consider that the atria are dynamic and adapt their function to maintain cardiac output in the presence of ventricular changes [34,35]. It is therefore possible that the RA dysfunction we observed in *MYH6* variant carriers is secondary to early RV dysfunction (i.e., during fetal life). Recent studies of surgically palliated HLHS patients have shown that atrial reservoir strain at the time of pre-Glenn echocardiograms correlates with mortality or the need for cardiac transplant prior to Fontan [36], and the authors hypothesized that atrial dysfunction may be secondary to changes in ventricular diastolic function. A notable distinction is that their findings showed that atrial reservoir strain was associated with low RV GLS [36], while we did not identify significant differences in RV strain indices between the groups. Furthermore, RV VSRed was not correlated with HR in *MYH6* variant carriers, which is inconsistent with ventricular stiffness and diastolic dysfunction as the primary pathology.

We did identify that RV GLS, VSs, and VSRs were positively correlated with HR in controls only, which could indicate a primary ventricular contractile deficit in *MYH6* variant carriers. Previous reports have found that RV VSs decreases with increasing HR in healthy children [37]. This could suggest that the relationship between RV strain and HR seen in our control patients may be an abnormal response. Unfortunately, studies are lacking that examine the relationship of RV strain and HR in the single-ventricle population, making it difficult to interpret our findings. 

### 4.4. Limitations

Our study did have some notable limitations. Assessing echocardiograms obtained prior to any surgical intervention is our best opportunity to examine native RA mechanics, as once staged palliation begins many factors impact both atria and overall cardiac function, potentially confounding the influence of *MYH6* variants. However, the retrospective nature of the study may have resulted in suboptimal echocardiogram views and variation in image quality over the years, including a lack of tricuspid color Doppler on many of the studies. While we were able to minimize some of these differences between the groups by control-matching based on approximate year of birth, this also limited our ability to match based on atrial restriction or account for the degree of tricuspid regurgitation. 

Our analysis also did not take into account differences in ventilation status between the groups. Mechanical ventilation impacts numerous aspects of cardiac hemodynamics, including right heart preload, afterload, and compliance [38,39], all of which must be considered when interpreting our RA and RV strain findings. Differences in the need for mechanical ventilation could also be a downstream effect of impaired cardiac function, though further studies are required to evaluate this hypothesis. Additionally, the small sample size may have limited the statistical power of our study and obscured potentially significant differences in RV function between the groups. Repeating these analyses in a larger cohort would help clarify this further.

It is also important to consider that in silico measures of *MYH6* variant pathogenicity cannot fully predict the functional impact. Further testing of specific *MYH6* variants using in vitro and animal models is required to delineate which variants have meaningful phenotypic consequences on cardiomyocyte contractility. A more extensive discussion of currently published genetic and functional data of *MYH6* variants, including those presented here, can be found in previous publications [7]. 

## 5. Conclusions

Our findings demonstrate atrial dysfunction associated with *MYH6* variants in HLHS. HLHS patients with *MYH6* variants have decreased atrial contractile strain, as well as an increase in reservoir and conduit strain with increasing HR. RV strain indices were not decreased in *MYH6* variant carriers, consistent with a primary atrial phenotype.

The confirmation of these findings in a larger cohort, along with longitudinal studies of atrial function in HLHS, are warranted to identify if RA dysfunction in *MYH6* variant carriers is evident throughout the lifetime of these patients and, if so, its relationship to decreased transplant-free survival. Such studies will further elucidate if atrial dysfunction could be an informative prognostic consideration in HLHS patients with *MYH6* variants and thus justify early genetic screening.

## Figures and Tables

**Table 2:** Patient characteristics.

	*MYH6* Variant n = 19 (%)	Control n = 37 (%)
*Sex*		
Male	9 (47.4)	20 (54.1)
Female	10 (52.6)	17 (45.9)
*Anatomy*		
AA/MA	10 (52.6)	19 (51.4)
AA/MS	2 (10.5)	4 (10.8)
AS/MS	7 (36.8)	14 (37.8)
RAS or IAS	7 (36.8)	12 (32.4)
*Respiratory Status*		
Mechanical ventilation	5 (26.3)	7 (18.9)
Room air or nasal cannula	12 (63.2)	29 (78.3)
Unknown	2 (10.5)	1 (2.7)
*Stage I shunt type*		
BTTS	10 (52.6)	19 (51.4)
RVPAC (Sano)	9 (47.4)	18 (48.6)

AA, aortic atresia; MA, mitral atresia; AS, atrial stenosis; MS, mitral stenosis; RAS, restrictive atrial septum; IAS, intact atrial septum; BTTS, Blalock–Thomas–Taussig shunt; and RVPAC, right ventricle to pulmonary artery conduit.

**Table 3:** RA and RV strain indices presented as median (IQR). Exact significance calculated using a two-sided Mann-Whitney U test.

	*MYH6* Variant (N = 19)	Control (N = 37)	*p*-Value
RA GLS (%)	18.2 (15.0, 24.1)	22.2 (14.9, 24.9)	0.263
RA AScd (%)	26.4 (20.7, 31.2)	27.0 (19.7, 34.4)	0.810
RA ASct (%)	−1.41 (−2.13, −0.25)	−3.53 (−5.53, −1.28)	0.008 **
RA ASr (%)	28.5 (21.2, 33.2)	31.0 (24.7, 37.5)	0.302
RA ASRr (%/s)	1.06 (0.78, 1.43)	1.23 (1.05, 1.55)	0.096
RA ASRct (%/s)	−1.26 (−1.55, −0.99)	−1.40 (−1.71, −1.13)	0.198
RV GLS (%)	−12.5 (−15.0, −11.3)	−12.5 (−14.4, −10.7)	0.683
RV VSs (%)	−14.3 (−16.3, −12.9)	−14.6 (−17.4, −11.4)	0.201
RV VSRs (%/s)	−0.89 (−0.99, −0.77)	−0.78 (−1.07, −0.66)	0.127
RV VSRed (%/s)	28.5 (21.2, 33.2)	31.0 (24.7, 37.5)	0.302
Heart rate (bpm)	145 (138, 157)	154 (142, 162)	0.283

RA, right atria; RV, right ventricle; GLS, global longitudinal strain; AScd, conduit atrial strain; ASr, reservoir atrial strain; ASct, active/contractile atrial strain; ASRr, reservoir atrial strain rate; ASRct, active/contractile atrial strain rate; VSs, systolic ventricular strain; VSRs, systolic ventricular strain rate; VSRed, early diastolic ventricular strain rate; and bpm, beats per minute. ** *p* < 0.01.

**Table 4:** Correlations of RA and RV strain indices with heart rate, presented with two-sided significance.

	*MYH6* Variant		Control	
	Pearson Correlation (R)	*p*-Value	Pearson Correlation (R)	*p*-Value
RA GLS (%)	0.268	0.267	−0.044	0.796
RA AScd (%)	0.469	0.043 *	−0.176	0.297
RA ASct (%)	−0.148	0.546	−0.220	0.190
RA ASr (%)	0.499	0.029 *	−0.102	0.547
RA ASRr (%/s)	0.368	0.121	−0.047	0.783
RA ASRct (%/s)	−0.235	0.332	0.274	0.100
RV GLS (%)	−0.001	0.997	0.325	0.050 *
RV VSs (%)	−0.069	0.779	0.419	0.010 *
RV VSRs (%)	−0.127	0.604	0.410	0.012 *
RV VSRed (%)	−0.100	0.685	−0.265	0.113

GLS, global longitudinal strain; AScd, conduit atrial strain; ASr, reservoir atrial strain; ASct, active/contractile atrial strain; ASRr, reservoir atrial strain rate; ASRct, active/contractile atrial strain rate; VSs, systolic ventricular strain; VSRs, systolic ventricular strain rate; and VSRed, early diastolic ventricular strain rate. * *p* < 0.05.

## Data Availability

Restrictions apply to the availability of these data. The data are restricted as they contain protected patient information.
